# Hospitalisation due to respiratory syncytial virus in a population-based cohort of older adults in Spain, 2016/17 to 2019/20

**DOI:** 10.2807/1560-7917.ES.2025.30.10.2400364

**Published:** 2025-03-13

**Authors:** Noelia Vera-Punzano, Camino Trobajo-Sanmartín, Ana Navascués, Aitziber Echeverria, Itziar Casado, Carmen Ezpeleta, Jesús Castilla, Iván Martínez-Baz

**Affiliations:** 1 Instituto de Salud Pública de Navarra, Pamplona, Spain; 2 Instituto de Investigación Sanitaria de Navarra (IdiSNA), Pamplona, Spain; 3 CIBER Epidemiología y Salud Pública (CIBERESP), Spain; 4 Clinical Microbiology Department, Hospital Universitario de Navarra, Pamplona, Spain

**Keywords:** Respiratory syncytial virus, RSV, Respiratory infection, Hospitalisation, Older adults, Risk factors, Cohort study, Vaccine

## Abstract

**Background:**

Respiratory syncytial virus (RSV) is a major cause of acute respiratory infection that can lead to complications in risk groups.

**Aim:**

We aimed to estimate the incidence of RSV hospitalisation in adults, determine the risk factors and characterise priority groups for prevention.

**Methods:**

This population-based cohort study included adults 60 years and older in Navarre, Spain, in seasons 2016/17 to 2019/20. We estimated the rate of RSV hospitalisation confirmed by PCR and evaluated risk factors using Poisson regression.

**Results:**

Within 642,622 person-years analysed, we detected 544 RSV hospitalisations (average annual rate: 84.7/100,000). The rate varied among seasons between 59.7 and 95.6 per 100,000. The rate ratio of hospitalisation was higher than 3 from the age of 75 years and around 7 in the 85–94 years age group compared with those aged 60–64 years. Nursing home residence, functional dependence, haematological cancer, chronic obstructive pulmonary disease (COPD), asthma, cardiovascular disease, severe obesity, diabetes and chronic kidney disease were independent risk conditions. Rate of RSV hospitalisation was higher than 300 per 100,000 among people with haematological cancer or nursing home residence, those aged ≥ 75 years with COPD or functional dependence, and those aged ≥ 85 years with asthma or cardiovascular disease. These groups represented 13.2% of all adults aged ≥ 60 years and 50.7% of their RSV hospitalisations. On average, these groups had one RSV hospitalisation per 307 person-years.

**Conclusion:**

Advanced age, in addition to nursing home residence, functional dependence and some comorbidities define priority groups for RSV vaccination.

**Key public health message**

**What did you want to address in this study and why?**

Respiratory syncytial virus (RSV) is a major cause of acute respiratory infection that may result in complications in the population over 60 years. Knowing how often this infection requires hospitalisation and what the risk factors are, is essential to plan preventive measures and recommendations for the new RSV vaccines. We wanted to characterise the population groups with the highest priority for RSV prevention.

**What have we learnt from this study?**

On average over four seasons, RSV caused 84.7 hospitalisations per 100,000 people older than 60 years. The rate of hospitalisation due to RSV was considerable (> 300 per 100,000) among people in nursing homes or with haematological cancer, those aged 75 years and older with chronic pulmonary disease or functional impairments, and those aged 85 years and older with asthma or cardiovascular disease.

**What are the implications of your findings for public health?**

Our findings show that the rate of RSV hospitalisation varies considerably with age and presence of risk conditions. Non-pharmacological preventive measures should be strengthened around the adults we identified to be at highest risk, and they may be considered a priority target population for current and new vaccines against RSV.

## Introduction

Respiratory syncytial virus (RSV) is a leading cause of acute respiratory tract infection. The RSV infection may result in complications, especially among infants and adults older than 60 years or those with major chronic comorbidities [1-3]. The most frequent severe presentations are bronchiolitis in infants and pneumonia in older adults, which often lead to hospitalisations and can be fatal [4,5]. Its presentation in epidemic waves causes significant overload of the health system in winters [6]. The incidence of RSV disease in infants and young children has been estimated in several studies [4,7,8].

Recent systematic reviews estimated ca 787,000 annual RSV-related hospitalisations in older adults in high-income countries, with an in-hospital case fatality ratio of around 6–8%, increasing to 10% in high-risk adults [9,10]. However, the rate of hospitalisation from RSV in older adults has not been well established, because before the COVID-19 pandemic, only a few sites routinely screened for RSV in adults admitted to hospitals with respiratory infections, and after the COVID-19 pandemic, virus circulation has shown increased variability. The incidence and severity of RSV infection in adults have been related to older age, pre-existing comorbidities and other socio-demographic factors such as nursing home residence [9,11-14].

The treatment for RSV disease is mainly supportive [15]. Ribavirin treatment is limited to immunocompromised patients due to safety concerns [15,16]. Non-pharmacological preventive measures may be useful in preventing RSV infections, especially in high-risk people. However, compliance with these measures over time is difficult and their effects disappear as soon as the measures are suspended. In 2023 and 2024, three vaccines were approved in the European Union for prevention in the older adults (Abrysvo, Pfizer; Arexvy, GlaxoSmithKline; and mResvia, Moderna) [16-19]. Given the considerable price of these products, assessing the incidence of severe RSV disease in older adults and characterising the population groups at higher risk for severe outcomes due to RSV infection are essential to plan preventive measures and the possible introduction of these new vaccines.

In this study we aimed to quantify, in a well-defined population over four RSV seasons, the average incidence rate of hospital admissions due to RSV in adults older than 60 years, determine risk factors and characterise the population groups with the highest priority for RSV prevention.

## Methods

### Study design and setting

We performed a population-based cohort study from electronic medical records in the region of Navarre (ca 660,000 inhabitants; ca 165,000 people ≥ 60 years), Spain, where the Regional Health Service provides universal healthcare, free at the point of service. We defined four cohorts starting on 1 October of each RSV season from 2016/17 to 2019/20. Each cohort included all adults 60 years or older who had resided and been covered by the Navarre Health Service during the previous 12 months. Each cohort was linked with surveillance data of RSV hospitalisations from 1 October to 30 September of the following year. The four 12-month periods were joined in a pooled analysis to obtain average estimates.

### Outcome variables

The outcome of interest was hospitalisation due to RSV confirmed by reverse-transcription PCR (RT-PCR). The RSV surveillance was based on electronic reporting of laboratory-confirmed cases from all hospitals in Navarre. The hospital protocol established early detection and double swabbing, nasopharyngeal and pharyngeal, of all patients admitted to the hospital with acute respiratory symptoms. Respiratory samples were tested for influenza and RSV by RT-PCR (Allplex Flu + RSV, Seegene), which is a recommended test for RSV diagnosis and surveillance [20].

### Potential risk factors

As potential risk factors we considered socio-demographic variables and specific risk conditions. Socio-demographic variables included biological sex, 5-year age groups, country of birth (Spain or other), municipality of residence grouped as rural (< 10,000 inhabitants) or urban area (> 10,000 inhabitants), as well as the respiratory viral season (from 2016/17 to 2019/20).

Risk conditions at baseline were obtained from the electronic medical records and included: asthma, chronic obstructive pulmonary disease (COPD), cardiovascular disease, immunodeficiency (including HIV infection, transplant recipient and congenital immunodeficiency), diabetes mellitus, liver cirrhosis, chronic kidney disease, haematological cancer, non-haematological cancer, rheumatic disease, cerebrovascular disease, dementia, severe obesity (body mass index ≥ 40 kg/m^2^), nursing home residence, and functional dependence (Barthel index < 40). Chronic comorbidities were codified according to the International Classification of Primary Care, Second Edition [21].

All patient information was linked using an individual identification number, and the database was anonymised before the analysis.

### Statistical analysis

The average annual incidence rate of RSV hospitalisation was calculated for the pool of the four study periods by dividing the total number of cases by the sum of the study population at the beginning of each period. The incidence rate of RSV hospitalisation per 100,000 people was calculated for each category of the variables analysed. We stratified these incidence rates of hospitalisation by sex and age category (60–74, 75–84 and ≥ 85 years). We evaluated the association of potential risk factors and RSV hospitalisation in crude and multivariate analyses, using Poisson regression models to obtain crude and adjusted rate ratios (RRs) with their 95% confidence intervals (CIs) and p values. Multivariate analyses were adjusted by sex, 5-year age groups, country of birth (Spain/other), rural/urban residence, respiratory viral season, and presence of each risk condition as separate variables. We considered p values lower than 0.05 statistically significant.

Finally, we characterised the priority population groups with annual risk for RSV hospitalisation higher than 300 per 100,000 by combination of age group and risk conditions. We compared this selection with that proposed in some reports which recommended vaccination for all people aged 75 years and older [22,23]. The comparison of target populations was performed in terms of number of persons divided by the number of RSV hospitalisations as indicator of the potential balance of a preventive intervention. We conducted the analyses using Stata version 17. Scripts are available in the Supplement.

## Results

### Average annual incidence rate of respiratory syncytial virus hospitalisation

During the four seasons studied, people 60 years and older accounted for 642,622 person-years and 544 hospitalisations due to RSV, which represented an average annual rate of 84.7 per 100,000 inhabitants. The annual number of RSV hospitalisations per season ranged between 95 (59.7/100,000) in the 2017/18 season and 155 cases (95.6/100,000) in the 2018/19 season (p = 0.001).

The average annual rate of hospitalisation did not differ significantly between males and females (92.2 vs 78.4/100,000; p = 0.058), but differences by age were relevant (p < 0.001). People 75 years and older were 38.6% of the study population, but led to 76.1% (n = 414) RSV hospitalisations. The rate of RSV hospitalisation was higher than 100 per 100,000 in adults 75 years and older, especially in the age groups 85–89 years (243.9/100,000) and 90–94 years (268.7/100,000). The rate of RSV hospitalisation was higher in the native than the foreign-born population (87.1 vs 23.7/100,000; p = 0.002). People residing in rural areas had a higher rate of RSV hospitalisation than urban residents (105.4 vs 68.9/100,000; p < 0.001). The rates of RSV hospitalisation were 18.8 and 131.1 per 100,000 (p < 0.001) among people without and with any risk condition, respectively. The conditions associated with the highest rates of RSV hospitalisation were haematological malignancies (399.0/100,000), nursing home residence (366.7/100,000), functional dependence (348.1/100,000) and COPD (269.7/100,000). The rate of RSV hospitalisations was also higher than 200 per 100,000 among patients with asthma, cardiovascular disease, chronic kidney disease and dementia (Table 1).

**Table 1:** Average annual rate of hospitalisation due to respiratory syncytial virus in the population ≥ 60 years, by socio-demographic variables and risk conditions, Navarre, Spain, 2016/17–2019/20 seasons (n = 642,622 person-years; n = 544 hospitalisations)

	Person-years	Hospitalisation		Rate per 100,000	p value
		n	% of hospitalised		
Sex					
Male	291,691	269	49.4	92.2	0.058
Female	350,931	275	50.6	78.4	
Age group, years					
60–64	147,454	28	5.1	19.0	< 0.001
65–69	129,937	44	8.1	33.9	
70–74	116,901	58	10.7	49.6	
75–79	85,850	89	16.4	103.7	
80–84	74,833	114	21.0	152.3	
85–89	54,940	134	24.6	243.9	
90–94	25,304	68	12.5	268.7	
≥ 95	7,403	9	1.7	121.6	
Country of birth					
Spain	617,334	538	98.9	87.1	0.002
Other	25,288	6	1.1	23.7	
Residence area					
Rural	278,065	293	53.9	105.4	< 0.001
Urban	364,557	251	46.1	68.9	
Risk conditions					
None	266,619	51	9.4	19.1	< 0.001
Any	376,003	493	90.6	131.1	
Individual risk conditions^a^					
Asthma	33,698	68	12.5	201.8	< 0.001
COPD	56,733	153	28.1	269.7	< 0.001
Cardiovascular disease	121,107	259	47.6	213.9	< 0.001
Immunodeficiency	3,458	4	0.7	115.7	0. 531
Diabetes	115,743	173	31.8	149.5	< 0.001
Liver cirrhosis	24,773	24	4.4	96.9	0.494
Chronic kidney disease	62,278	129	23.7	207.1	< 0.001
Haematological cancer	6,266	25	4.6	399.0	< 0.001
Non-haematological cancer	110,320	125	23.0	113.3	0.099
Rheumatic disease	9,807	14	2.6	142.8	0.110
Cerebrovascular disease	33,881	64	11.8	188.9	< 0.001
Dementia	22,436	50	9.2	222.9	< 0.001
Severe obesity	13,456	22	4.0	163.5	0.056
Nursing home residence	13,635	50	9.2	366.7	< 0.001
Functional dependence	18,387	64	11.8	348.1	< 0.001
Season					
2016/17	155,839	148	27.2	95.0	0.001
2017/18	159,099	95	17.5	59.7	
2018/19	162,050	155	28.5	95.6	
2019/20	165,634	146	26.8	88.1	
Total	642,622	544	100.0	84.7	NA

COPD: chronic obstructive pulmonary disease; NA: not applicable.

**^a^** More than one condition may be present in a person.

**Figure 1:**
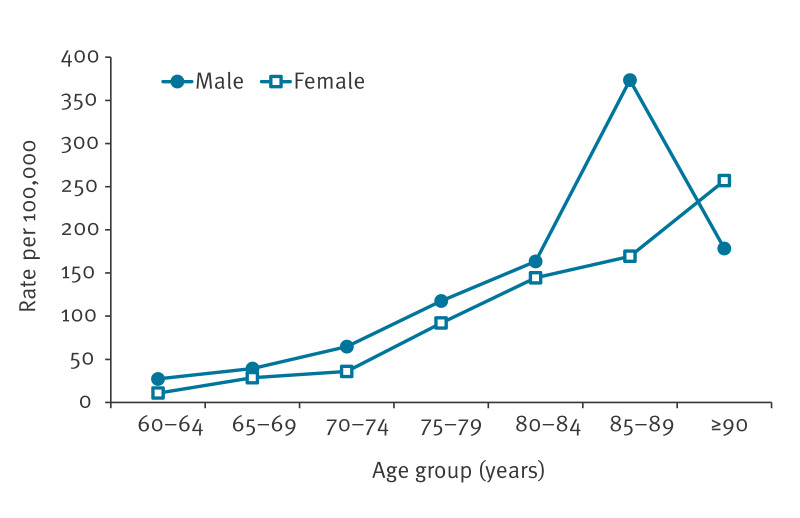
Average annual rates of hospitalisation due to respiratory syncytial virus infection in the population ≥ 60 years, by age group and sex, Navarre, Spain, 2016/17–2019/20 seasons (n = 642,622 person-years; n = 544 hospitalisations)

The rates of hospitalisation due to RSV increased with age in both sexes (p < 0.001), except in males 90 years and older who represented few hospitalised cases (Figure 1).

The combination of age category and the presence of each specific risk condition led to important differences in the average annual rate of RSV hospitalisation. The rate was lower than 40 per 100,000 in people younger than 85 years without risk conditions. However, in some age categories, people with haematological cancer, nursing home residence, functional dependence, COPD, asthma, cardiovascular diseases and diabetes reached average rate of RSV hospitalisation higher than 300 per 100,000 (Table 2).

**Table 2:** Average annual rates of hospitalisation due to respiratory syncytial virus in the population ≥ 60 years, by age category, socio-demographic variables and risk conditions, Navarre, Spain, 2016/17–2019/20 seasons (n = 642,622 person-years; n = 544 hospitalisations)

	60–74 years			75–84 years			≥ 85 years		
	Hospi- talised n	Rate per 100,000	p value	Hospi- talised n	Rate per 100,000	p value	Hospi- talised n	Rate per 100,000	p value
Sex									
Male	81	42.1	0.002	97	137.9	0.250	91	313.3	0.002
Female	49	24.3		106	117.3		120	204.8	
Country of birth									
Spain	125	33.5	0.434	203	128.7	0.999	210	242.3	0.395
Other	5	23.5		0	0.0		1	103.3	
Residence area									
Rural	67	41.1	0.019	112	155.2	0.004	114	266.5	0.130
Urban	63	27.2		91	102.8		97	216.2	
Risk conditions									
None	13	6.6	< 0.001	17	34.1	< 0.001	21	112.7	< 0.001
Any	117	59.7		186	167.8		190	275.3	
Individual risk conditions^a^									
Asthma	19	101.6	< 0.001	28	284.2	< 0.001	21	408.1	0.013
COPD	53	161.7	< 0.001	57	357.3	< 0.001	43	537.1	< 0.001
Cardiovascular disease	52	100.1	< 0.001	101	247.8	< 0.001	106	373.2	< 0.001
Immunodeficiency	3	110.3	0.036	1	150.6	0.860	0	0.0	0.999
Diabetes	39	66.2	< 0.001	75	200.2	0.003	59	303.9	0.043
Liver cirrhosis	10	56.8	0.079	12	214.4	0.063	2	127.1	0.259
Chronic kidney disease	23	126.5	< 0.001	48	208.5	0.001	58	275.1	0.362
Haematological cancer	10	324.3	< 0.001	9	432.5	< 0.001	6	545.0	0.044
Non-haematological cancer	35	54.8	0.001	50	154.8	0.108	40	281.6	0.279
Rheumatic disease	4	66.1	0.161	7	275.7	0.038	3	245.9	0.971
Cerebrovascular disease	7	60.4	0.104	30	248.7	< 0.001	27	264.0	0.610
Dementia	5	220.6	< 0.001	19	229.4	0.008	26	218.7	0.599
Severe obesity	9	102.8	0.001	10	285.6	0.009	3	250.2	0.946
Nursing home residence	6	307.9	< 0.001	16	387.3	< 0.001	28	370.6	0.017
Functional dependence	4	229.8	< 0.001	22	453.9	< 0.001	38	322.1	0.054
Season									
2016/17	36	37.9	0.330	50	125.6	0.325	62	294.2	0.001
2017/18	25	25.6		40	100.9		30	137.9	
2018/19	30	30.1		59	146.8		66	297.8	
2019/20	39	38.3		54	131.6		53	233.9	
Total	130	33.0	NA	203	126.3	NA	211	240.7	NA

COPD: chronic obstructive pulmonary disease; NA: not applicable.

**^a^** More than one condition may be present in a person.

### Risk factors for respiratory syncytial virus hospitalisation

The multivariate analysis showed that the incidence of hospitalisation due to RSV was strongly associated with the increased age. Compared with people aged 60–64 years, the rate increased 2-, 3-, 4- and 6- fold in people older than 70, 75, 80 and 85 years, respectively. The rate of RSV hospitalisation did not differ significantly by sex and country of birth in the adjusted analysis. Urban residents had lower rate of RSV hospitalisation than rural residents (RR = 0.73; 95% CI: 0.62–0.87). Seasonal variability in the rate was occasionally observed, since one of the four studied seasons (2017/18) presented a statistically significant lower rate of RSV hospitalisation (Table 3).

**Table 3:** Association between socio-demographic factors and hospitalisation due to respiratory syncytial virus in the adults ≥ 60, Navarre, Spain, 2016/17–2019/20 seasons (n = 642,622 person-years; n = 544 hospitalisations)

	Crude analysis			Adjusted analysis^a^		
	Rate ratio	95% CI	p value	Rate ratio	95% CI	p value
Sex						
Male	1	Reference		1	Reference	
Female	0.85	0.72–1.01	0.058	0.93	0.77–1.11	0.413
Age group, years						
60–64	1	Reference		1	Reference	
65–69	1.78	1.11–2.86	0.017	1.56	0.97–2.51	0.066
70–74	2.61	1.66–4.10	< 0.001	2.00	1.27–3.15	0.003
75–79	5.46	3.57–8.35	< 0.001	3.70	2.40–5.68	< 0.001
80–84	8.02	5.31–12.13	< 0.001	4.74	3.10–7.23	< 0.001
85–89	12.84	8.55–19.30	< 0.001	6.86	4.49–10.47	< 0.001
90–94	14.15	9.11–21.98	< 0.001	7.26	4.58–11.53	< 0.001
≥ 95	6.40	3.02–13.57	< 0.001	3.09	1.43–6.68	0.004
Country of birth						
Spain	1	Reference		1	Reference	
Other	0.27	0.12–0.61	0.002	0.56	0.25–1.25	0.155
Residence area						
Rural	1	Reference		1	Reference	
Urban	0.65	0.55–0.77	< 0.001	0.73	0.62–0.87	< 0.001
Season						
2016/17	1	Reference		1	Reference	
2017/18	0.63	0.49–0.81	< 0.001	0.65	0.50–0.84	0.001
2018/19	1.01	0.80–1.26	0.951	1.02	0.82–1.28	0.832
2019/20	0.93	0.74–1.17	0.523	0.94	0.74–1.18	0.579

CI: confidence interval; COPD: chronic obstructive pulmonary disease.

^a^ Analysis adjusted for all the variables in the table and the following risk conditions as separate variables: asthma, COPD, cardiovascular disease, immunodeficiency, diabetes mellitus, liver cirrhosis, chronic kidney disease, haematological cancer, non-haematological cancer, rheumatic disease, cerebrovascular disease, dementia, severe obesity, functional dependence and nursing home residence.

**Figure 2:**
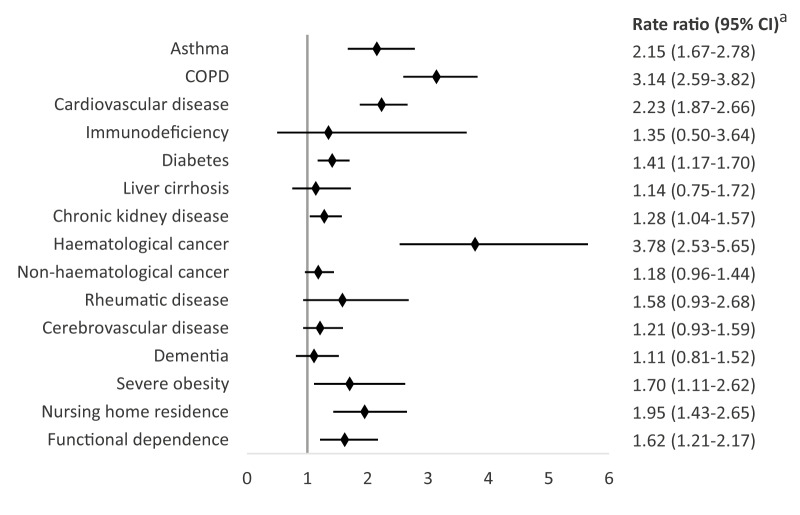
Association between risk conditions and respiratory syncytial virus hospitalisation in adults ≥ 60 years, Navarre, Spain, 2016/17–2019/20 seasons (n = 642,622 person-years; n = 544 hospitalisations)

CI: confidence interval; COPD: chronic obstructive pulmonary disease.

^a^ Rate ratio and 95% confidence interval obtained from a Poisson regression analysis adjusted for sex, 5- year age group, immigrant status, residence area, respiratory viral season and all risk conditions shown in the figure.

**Figure 3:**
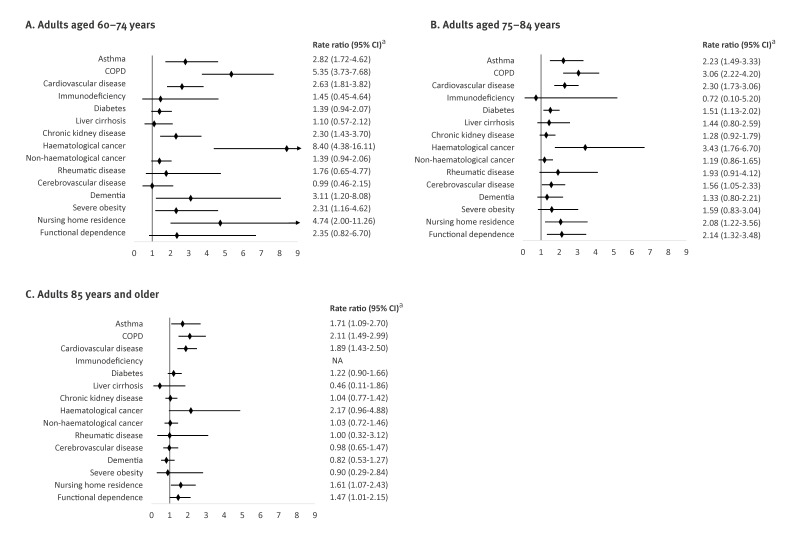
Association between risk conditions and respiratory syncytial virus hospitalisation in different adult age groups, Navarre, Spain, 2016/17 to 2019/20 seasons (n = 642,622 person-years; n = 544 hospitalisations)

CI: confidence interval; COPD: chronic obstructive pulmonary disease; NA: not applicable.

^a^ Rate ratio and 95% confidence interval obtained from a Poisson regression analysis adjusted for sex, 5-year age group, immigrant status, residence area, respiratory viral season and all risk conditions shown in the figure.

In the overall multivariate analysis, we observed increased rates of RSV hospitalisation in patients with haematological cancer (RR = 3.78; 95% CI: 2.53–5.65), COPD (RR = 3.14; 95% CI: 2.59–3.82), cardiovascular disease (RR = 2.23; 95% CI: 1.87–2.66), asthma (RR = 2.15; 95% CI: 1.67–2.78), severe obesity (RR = 1.70; 95% CI: 1.11–2.62), diabetes (RR = 1.41; 95% CI: 1.17–1.70), chronic kidney disease (RR = 1.28; 95% CI: 1.04–1.57), nursing home residence (RR 1.95; 95%CI 1.43–2.65) and functional dependence (RR = 1.62; 95% CI: 1.21–2.17) (Figure 2). The adjusted RR of RSV hospitalisation for each comorbidity showed differences among age categories. In general, RRs were high in the younger age category (60–74 years) and decreased or disappeared in older age categories (Figure 3).

### Characterisation of groups with higher risk of hospitalisation

The average annual rate of hospitalisation for RSV among the population aged 75 years and older was 166.7 per 100,000, i.e. one RSV hospitalisation per 600 persons. Combining the age categories and risk conditions we defined the population groups with an average annual rate of RSV hospitalisation higher than 300 per 100,000. These population groups included all people 60 years and older with haematological cancer or nursing home residence, as well as people aged 75 years and older with COPD or functional dependence, and people aged 85 years and older with asthma or cardiovascular disease. These groups represented 13.2% of all adults aged 60 years and older and 50.7% of their RSV hospitalisations. On average, these groups had one RSV hospitalisation per 307 person-years.

## Discussion

The present study showed that the rate of hospitalisation due to RSV in people older than 60 years varied considerably with age and presence of risk conditions. On average over four RSV seasons, the rate of RSV hospitalisations was 84.7 per 100,000, but reached more than 200 RSV hospitalisations per 100,000 among nursing home residents, people 85 years and older, those with functional dependence, chronic respiratory diseases, cardiovascular disease, kidney disease, haematological cancer or dementia.

Since the rate of RSV hospitalisation among people older than 60 years was very different depending on their socio-demographic characteristics and risk conditions, these findings help to define target population groups to be especially protected with non-pharmacological preventive measures (avoiding contact with persons with respiratory symptoms, maintaining physical distance, using facemasks, etc.) and to be prioritised in a gradual introduction of the new vaccines against RSV [16-18].

The overall rate of RSV hospitalisations for the studied population was close to that reported by Zhou et al. in the United States for those 65 years and older (86.1/100,000) [24]. In contrast, a study that analysed the hospital discharge database of Spain estimated 23.7 annual hospitalisations per 100,000 population aged 65 years or older [12]. This source appears to largely underestimate the number of RSV hospitalisations compared with our specific surveillance of RT-PCR-confirmed cases. In some northern European countries, estimates obtained by time series analysis led to a wide range of RSV hospitalisation rates in older people [25], our estimates being included in this range.

The rate of RSV hospitalisation increased progressively with age, reaching the highest rate in the 85–94 years age groups with more than 200 RSV hospitalisations per 100,000. This finding is consistent with other studies that described an upward trend with increasing age [9,12,13,26,27]. Immunosenescence may explain the increased risk of severe RSV outcomes in the older age groups [13,28], and that most of the RSV hospitalisations in adults affected patients 75 years and older [26].

Several major chronic conditions were associated with a significant increased risk of hospitalisation due to RSV infection, since the risk of RSV infection may be higher and the disease more severe in patients with underlying chronic conditions, which can be decompensated by the respiratory infection. Haematological cancer was the comorbidity that most increased the risk, multiplying it by 3.78 on average, which is consistent with results reported by Wyffels et al., who found a risk of RSV hospitalisation five times higher in these patients [27]. Another important risk factor for RSV hospitalisation in the present study was COPD, which may be because COPD can decompensate due to respiratory tract infection. In a prospective study, RSV infection accounted for 11% of hospitalisations for COPD [29]. Cardiovascular disease was the most frequent major chronic condition, which was present in 18.8% of the people included in the study cohort and 47.6% of patients hospitalised for RSV. The risk of RSV hospitalisation was doubled in patients with cardiovascular disease, as RSV infection may worsen conditions such as congestive heart failure or ischemic cardiovascular disease. Underlying cardiovascular disease has been associated with hospitalisation in 45–63% of adults with confirmed RSV [30].

Other risk conditions independently associated with RSV hospitalisation were functional dependence, asthma, severe obesity, diabetes and chronic kidney disease. All these conditions may be considered to define possible target populations for the RSV vaccination.

Immunodeficiency was not associated with a statistically significant higher risk of RSV hospitalisation in the present study, which may be explained by the small number of patients with these conditions and the reinforced application of preventive measures around them.

Our results are consistent with a systematic literature review concluding that immunosenescence and risk conditions contribute to a similar extent to an elevated risk of severe RVS disease [13]. We found that most risk conditions had a more pronounced influence on the rate of RSV hospitalisation in the younger age groups. However, as the risk also increased with age, the final risk was higher in older people than in younger people with similar risk conditions.

People residing in nursing homes presented a twofold higher rate of RSV hospitalisation than other people with similar characteristics who did not live in these facilities. The augmented risk of hospitalisation might be due to increased transmission or outbreaks in these facilities due to the close contact with caregivers and other vulnerable persons. A systematic review of the burden of respiratory infections in adults in long-term care facilities found an RSV incidence ranging from 1.1% to 13.5% [31]. This highlights the convenience of preventive measures such as facemask use, hand hygiene and avoiding contact between symptomatic persons in these facilities, as they have proven useful in preventing transmission during the COVID-19 pandemic [32]. The results support the inclusion of nursing home residents among the population groups to be prioritised for vaccination against RSV.

Residents in urban areas presented a lower rate of RSV hospitalisation than residents in rural areas. Seeking medical care earlier and having better access to hospitals could explain this difference.

Multivariate analyses showed no association between sex or country of birth and the risk of RSV hospitalisation. The lack of difference by sex was consistent with the result of another study [27]. Statistically significant differences in the rate of RSV hospitalisation were observed only in one of the four analysed seasons, which suggest a relatively stable incidence over the seasons, but with some possible variability.

New vaccines have been developed to prevent severe cases of RSV infection in adults [17,18,20]. At first, the limited availability of vaccines, their high price and the large number of people potentially eligible for vaccination make it necessary to prioritise the target population for vaccination. 

Strengths of this study are the population-based cohort design over four RSV seasons, the protocol for testing all hospitalised patients with severe acute respiratory diseases, and the inclusion of only laboratory-confirmed cases by RT-PCR admitted to the hospital due to complications of the RSV infection. The analysis of four consecutive RSV seasons before the COVID-19 pandemic, provides average results and rules out the bias due to any atypical season. Under the mentioned conditions, RSV hospitalisations are a valid source of information on severe RSV disease in the population, while data of primary healthcare consultations or mortality due to RSV are more prone to bias due to under-detection of cases [33].

Our study also has several limitations. RSV circulation can vary considerably among countries or seasons, so generalisations should be made with caution. The data on pre-existing chronic diseases were obtained from the electronic medical records of primary healthcare at the beginning of each season, so some misclassification is possible. Nevertheless, the validity of diagnoses from this source was evaluated in other studies in Navarre [34]. The present study has evaluated only one aspect for the decision of introducing a new vaccine; therefore, studies about the incidence of other RSV outcomes (such as medical consultations, intensive care unit admission and mortality), studies of vaccine effectiveness and economic evaluation studies are also necessary. It has been recommended to test more than one specimen from each patient to increase the RSV detection [35]. Our protocol recommended double swabbing (nasopharyngeal and pharyngeal), which partially corrects limitations in detection sensitivity.

## Conclusions

Respiratory syncytial virus infections are responsible for an important number of hospitalisations in older adults during annual epidemics, but the rate of RSV hospitalisation varies considerably with age and presence of risk conditions. According to our results, a first priority population group for preventive measures, because of their very high rate of RSV hospitalisation (> 0.3% per year), includes people 60 years and older with haematological cancer or nursing home residence, people older than 74 years with COPD or functional dependence, and people older than 84 years with asthma or cardiovascular disease. This strategy would involve immunising only 13% of people aged 60 years and older, but would address half of RSV hospitalisations in this age group. The prevention of these hospitalisations would appreciably reduce the likelihood of hospital resources being overwhelmed during the weeks with high RSV circulation and would probably prevent a high proportion of RSV-related deaths. Our findings provide useful information to estimate the potential impact of different strategies for the introduction of RSV vaccines in older adults. Non-pharmacological preventive measures should be strengthened around them, and they may be considered a priority target population for new vaccines against RSV.

## Supplementary Data

351000 Supplement
