# Supplementary material for: Hospitalisation due to respiratory syncytial virus in a population-based cohort of older adults in Spain, 2016/17 to 2019/20
Source: Euro Surveill. 2025 Mar 13;30(10):2400364. doi: 10.2807/1560-7917.ES.2025.30.10.2400364 (PMC11912144; doi:10.2807/1560-7917.ES.2025.30.10.2400364)
Supplement: Supplement [file 24-00364_CASTILLA_Supplement.pdf]

Supplementary Material

This supplementary material is hosted by Eurosurveillance as supporting information alongside the article "Hospitalisation due to respiratory syncytial virus in a population-based cohort of older adults in Spain, 2016/17 to 2019/20", on behalf of the authors, who remain responsible for the accuracy and appropriateness of the content. The same standards for ethics, copyright, attributions and permissions as for the article apply. Supplements are not edited by Eurosurveillance and the journal is not responsible for the maintenance of any links or email addresses provided therein.

Definition of the variables

Variable description in the text	Variable name in the model	Possible values
RSV hospitalisation	hospital	Absence (0), Presence (1)
Sex	sex	Male (0), Female (1)
5-year age group	Age	60-64 (0), 65-69 (1), 70-74 (2), 75-79 (3), 80-84 (4), 85-89 (5), 90-94 (6), ≥ 95 (7) years old
Country of birth	foreign	Spain (0), Other (1)
Rural/urban residence	urban	Rural (0), Urban (1)
Respiratory viral season	season	2016/17 (0), 2017/18 (1), 2018/19 (2), 2019/20 (3)
Presence of risk conditions		
Any risk conditions	any_risk	Absence (0), Presence (1)
Asthma	asthma	Absence (0), Presence (1)
Chronic obstructive pulmonary disease (COPD)	copd	Absence (0), Presence (1)
Cardiovascular disease	cv	Absence (0), Presence (1)
Immunodeficiency (including HIV infection, transplant recipient and congenital immunodeficiency)	inmuno	Absence (0), Presence (1)
Diabetes mellitus	diabetes	Absence (0), Presence (1)
Liver cirrhosis	cirrhosis	Absence (0), Presence (1)
Chronic kidney disease	kidney	Absence (0), Presence (1)
Haematological cancer	c_hema	Absence (0), Presence (1)
Non-haematological cancer	c_nohema	Absence (0), Presence (1)
Rheumatic disease	rheuma	Absence (0), Presence (1)

Cerebrovascular disease	stroke	Absence (0), Presence (1)
Dementia	dementia	Absence (0), Presence (1)
Severe obesity (body mass index ≥ 40 kg/m2)	obes	Absence (0), Presence (1)
Nursing home residence	ltcf	Absence (0), Presence (1)
Functional dependence (Barthel index < 40)	depen	Absence (0), Presence (1)

Program code (Stata versión 17)

Average anual rate of hospitalisation due to RSV

```
foreach var of varlist sex age foreign urban any_risk asthma copd cv immuno diabetes ///
> cirrhosis kidney c_hema c_nohema rheuma stroke dementia ///
> obes ltcf depen season {tab `var' hospital,m ro chi }
```

Association between socio-demographic factors and hospitalisation due to RSV

Crude Poisson regression models

```
poisson hospital sex, irr
poisson hospital i.age, irr
poisson hospital foreign, irr
poisson hospital urban, irr
poisson hospital asthma, irr
poisson hospital copd, irr
poisson hospital cv, irr
poisson hospital immuno, irr
poisson hospital diabetes, irr
poisson hospital cirrhosis, irr
poisson hospital kidney, irr
poisson hospital c_hema, irr
poisson hospital c_nohema, irr
```

poisson hospital rheuma, irr

poisson hospital stroke, irr

poisson hospital dementia, irr

poisson hospital obes, irr

poisson hospital ltcf, irr

poisson hospital depen, irr

poisson hospital i.season, irr

Adjusted Poisson regression model

poisson hospital sex i.age foreign urban asthma copd cv immuno diabetes cirrhosis kidney c_hema c_nohema rheuma stroke dementia obes ltcf depen i.season, irr

Stratified analysis by age category

keep if sex==0

foreach var of varlist age {tab `var' hospital,m ro chi }

keep if sex==1

foreach var of varlist age {tab `var' hospital,m ro chi }

Stratified analysis by age category

keep if age==0 | age==1 | age==2

foreach var of varlist sex age foreign urban asthma copd cv immuno diabetes cirrhosis ///

> kidney c_hema c_nohema rheuma stroke dementia ///

> obes ltcf depen season {tab `var' hospital,m ro chi }

Adjusted Poisson regression model

poisson hospital sex i.age foreign urban asthma copd cv immuno diabetes cirrhosis kidney c_hema c_nohema rheuma stroke dementia obes ltcf depen i.season, irr

keep if age==3 | age==4

foreach var of varlist sex age foreign urban asthma copd cv immuno diabetes cirrhosis ///

> kidney c_hema c_nohema rheuma stroke dementia ///

```
> obes ltcf depen season {tab `var' hospital,m ro chi }
```

Adjusted Poisson regression model

```
poisson hospital sex i.age foreign urban asthma copd cv immuno diabetes cirrhosis kidney c_hema c_nohema rheuma stroke dementia obes ltcf depen i.season, irr
```

```
keep if age==5 | age==6 | age==7

foreach var of varlist sex age foreign urban asthma copd cv immuno diabetes cirrhosis ///

> kidney c_hema c_nohema rheuma stroke dementia ///

> obes ltcf depen season {tab `var' hospital,m ro chi }
```

Adjusted Poisson regression model

```
poisson hospital sex i.age foreign urban asthma copd cv immuno diabetes cirrhosis kidney c_hema c_nohema rheuma stroke dementia obes ltcf depen i.season, irr
```
